# Female Soccer Players’ In-Season Weekly Training Load and Intensity: Comparison between National League’s Top and Bottom-Half Ranked Teams

**DOI:** 10.5114/jhk/189657

**Published:** 2024-12-06

**Authors:** Eero H. J. Savolainen, Johanna K. Ihalainen, Simon Walker

**Affiliations:** 1 Faculty of Sport and Health Sciences, University of Jyväskylä, Jyväskylä, Finland.; 2 Finnish Institute of High Performance Sport KIHU, Jyväskylä, Finland.; 3 Neuromuscular Research Center, University of Jyväskylä, Jyväskylä, Finland.

**Keywords:** soccer, women, external load, internal load, microcycle

## Abstract

This study aimed to (1) quantify national-level female soccer players’ accumulated microcycle (weekly) training load (accumulated absolute value) and intensity (value relative to duration), (2) investigate possible differences in training load and intensity between teams in the league’s top- and bottom-half. Sixty-three females from six teams in the highest league participated in the study. Participants were divided into top-half (TH, n = 28) and bottom-half (BH, n = 35) groups based on their team’s league ranking. Players’ external and internal training load and intensity were monitored in all on-field training sessions and league matches during a three-week observation period (690 data samples) using the Polar Team Pro system. There were no differences between groups in the number of weekly training sessions or total duration. Accumulated external and internal load were highly similar between groups. Instead, the TH group reached significantly higher values than the BH group in multiple external intensity variables, such as total distance per minute and the number of low-, moderate- and high-intensity accelerations per minute (p = 0.004–0.001). There were no differences in the mean heart rate between groups. The TH group’s training external intensity was also closer to match intensity in multiple variables, such as total distance and the number of low- and moderate-intensity accelerations and decelerations compared to the BH group (p = 0.029–0.001). These findings suggest that more successful teams achieved higher external intensity for the same internal response, and their average external training intensity was closer to match demands. Thus, it seems plausible that TH players had better physical qualities and their training prepared them better for matches than BH.

## Introduction

Training load monitoring is used extensively in soccer. In elite female soccer, teams use training load monitoring to improve planning of training, assess players’ performance, improve match management, and promote players’ rehabilitation (Luteberget et al., 2021). Training load can be divided into external and internal training load, where the external training load is the physical work performed during the training session or competition, while the internal training load represents psychophysiological responses to the external load that provides the stimulus for training adaptation (Impellizzeri et al., 2019). Thus, a similar internal load can lead to a considerably different external load generated by players in soccer (Mäkiniemi et al., 2023), being dependent upon the players’ physical qualities (Savolainen et al., 2023).

A recent review on monitoring of training load in soccer highlighted the need for research on training load/match load monitoring in female players (Teixeira et al., 2021). Since then, other studies on female players have been published, but those have mainly focused on quantifying the match load (Mäkiniemi et al., 2023) or investigating microcycle periodization (Chamera et al., 2023; Doyle et al., 2022; Karlsson et al., 2023; Krawczyk et al., 2022; Romero-Moraleda et al., 2021), while less focus has been placed on the accumulated training load during the microcycle (Diaz-Seradilla et al., 2022; McFadden et al., 2020). In a recent review, Costa et al. (2022) showed that most studies on training load of female soccer players were observational studies conducted with only one group (i.e., one team), thus the results of these studies reflect the training approach of one specific coaching staff and cannot be readily generalized.

From the available data, female players have shown that the average external training load is highest in the pre-season or the first part of the in-season and decreases towards the end of the in-season (Karlsson et al., 2023; Mara et al., 2015). Within the in-season, professional female players’ microcycle (i.e., weekly) accumulated total running distance varies between 22 and 24 km, from which 450–550 m is above the 16 km/h speed threshold and 300–450 m occurs above the 19 km/h speed threshold (Diaz-Seradilla et al., 2022). Nevertheless, even higher total distances have been reported in college players, ranging between 20 and 30 km per microcycle (McFadden et al., 2020). In previous literature, the session rating of perceived exertion (sRPE) has been used to quantify female soccer players’ accumulated microcycle internal load. In Portuguese first division players, the sRPE has been shown to vary between ~1500 and 3000 arbitrary units (AU) (Gonçalves et al., 2021).

As far as the authors are aware, there are no previous studies that have compared training load (i.e., accumulated absolute value) or intensity (i.e., accumulated values relative to duration) (Gaudino et al., 2015) between teams in the same league divergent for success. Instead, previous studies conducted with male players have examined the effects of players’ age and their performance level on the accumulated training load and intensity (Coppalle et al., 2021; Hannon et al., 2021; Houtmeyers et al., 2020). It has been shown that the weekly microcycle accumulated external training load in male academy players increases in-line with age (Hannon et al., 2021). Direct comparison of the training load and intensity between male professional first team players and players of the U19 team of the same club has shown mixed results (Coppalle et al., 2021; Houtmeyers et al., 2020), however, Houtmeyers et al. (2020) showed that U19 players reached higher total distance at low-speed during the weekly microcycle, yet the intensity of the external load variables was lower in U19 players compared to first team players. Instead, Coppalle et al. (2019) showed that U19 players reached higher external and internal load compared to first team players. Thus, the effect of the playing level on the accumulated training load remains unclear, even in male professional soccer, while previous studies have only investigated the effects of playing level on match load in female soccer. Those studies have shown that match demands increase in-line with competition level (Vescovi et al., 2021), especially in terms of high-intensity running (Mohr et al., 2008), and that the external load is higher when playing against a similarly ranked team (Hewitt et al., 2014).

Given the lack of knowledge on the accumulated training load and intensity of female soccer players, especially studied across multiple teams, the aims of this study were to: 1) quantify national-level female soccer players’ accumulated microcycle training load and intensity, and 2) investigate whether there were differences in training load or intensity variables between teams in the top- and bottom-half of the league. We hypothesized that: 1) the microcycle’s accumulated training load and intensity would be similar to those reported previously in professional female players (Diaz-Seradilla et al., 2022; McFadden et al., 2020), and 2) neither training load nor intensity would differ between the league’s top- and bottom-ranked teams, since the effect of the playing level on the accumulated training load is unclear (Coppalle et al., 2021; Houtmeyers et al., 2020).

## Methods

### Participants

One-hundred and ten national-level players (defined according to McCay et al. (2022)) from six Finnish national-league teams volunteered for the study. Finnish national-league player status varies from professional to amateur, but the majority of players here were amateur or semi-professional. Sixty-three participants met the inclusion criteria, and their data were included in the final analyses. The inclusion criteria were: i) an out-field playing position, ii) the player participated in at least 80% of their team’s on-field training during the microcycle sessions (Oliveira et al., 2019), and iii) the player played at least 60 min in that microcycle’s league match (Stevens et al., 2017).

Participants were divided into two groups based on their team’s league ranking at the end of the season. In the top-half group (TH, n = 28, age 23.3 ± 4.7 years, 10 defenders, 11 midfielders, 7 attackers) the teams’ league rankings were between 1 and 5 (on average 2.0 ± 0.3 points per match during the season and 2.0 ± 0.6 during the observation period) and in the bottom-half group (BH, n = 35, age 21.5 ± 2.4, 12 defenders, 14 midfielders, 9 attackers) team rankings ranged from 6 to 10 (on average 1.0 ± 0.2 points per match during the season and 0.7 ± 0.7 during the observation period). All players provided written informed consent prior to participating in the study. The study was approved by the ethics committee of the University of Jyväskylä (approval code: 1064/13.00.04.00/2020; approval date: 04 September 2020) and conducted according to the Declaration of Helsinki (2013), except for registration in a database.

### Design and Procedures

**Figure 1:**
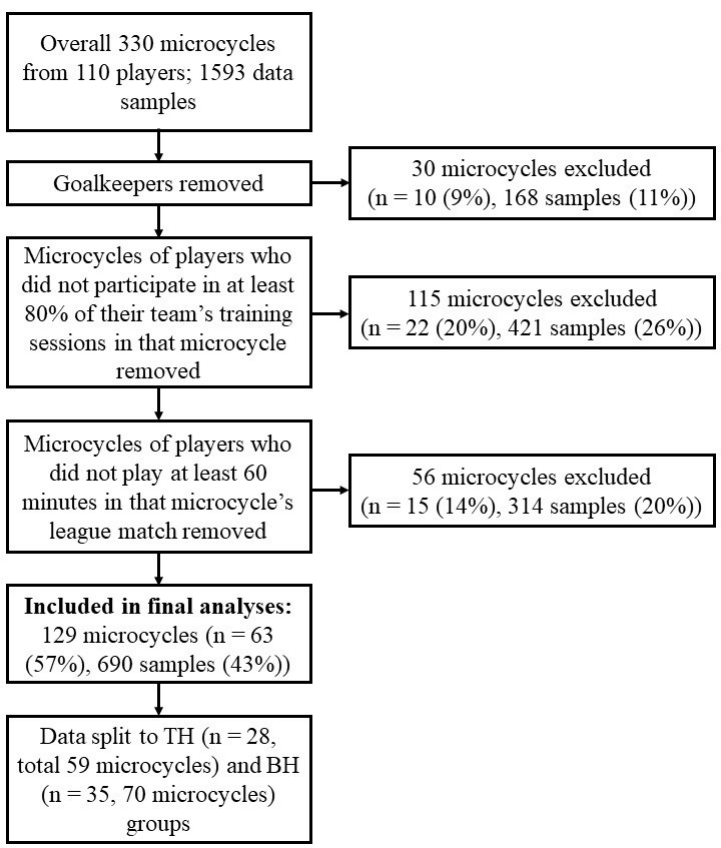
Study's data processing.

In this observational study, data were collected during three weeks at the beginning of the 2020 in-season. During the study period (microcycles), all teams had a league match at the weekend. During the observational period, one hundred and twenty-nine microcycles from 63 players (overall 690 data samples) fulfilling the inclusion criteria were used in the final analysis (Figure 1).

Weekly training load/intensity values used in analyses included data from all weekly on-field training sessions and from the league match. For training sessions, players were monitored throughout the entire session (including the on-field warm-up). Only training load data from on-field training sessions and matches were used; gym sessions were not included in this study. For matches, players were monitored from the start of the match until the final whistle, i.e., not including warm-up data, in accordance with Stevens et al. (2017). To standardize the match load, a 90-min value was obtained by dividing match load values by playing minutes and multiplying by 90. The match load was standardized to 90-min values to compare between the microcycle’s load and intensity to match values (Stevens et al., 2017).

### Measures

External and internal load were measured by the Polar Team Pro player tracking system (Polar Electro Oy, Kempele, Finland) with the Global Positioning System (GPS) sampled at 10 Hz, a triaxial accelerometer sampled at 200 Hz, a gyroscope, a magnetometer and a HR monitor. Data entered into the analyses were generated by accompanying Polar Team Pro online software. Good-to-moderate reliability (< 5% CV) and validity for total distance, linear running and team sport simulation circuit have been reported for the Polar Team Pro system (Huggins et al., 2020).

The following variables were used to represent the external microcycle load: total distance [m], distance covered in zones 1 (< 7 km/h), 2 (7–13 km/h), 3 (13–19 km/h), 4 (19–23 km/h) and 5 (> 23 km/h) [m] (FIFA, 2019), the number of low- (1–2 m/s^2^), moderate- (2–3 m/s^2^) and high-intensity (> 3 m/s^2^) accelerations and low- (−1 to −2) m/s^2^), moderate- (−2 to −3) m/s^2^) and high-intensity (<−3 m/s^2^) decelerations [n]. The following variables were used to represent the internal load: the average heart rate (HR) [%/ HR_max_], Edwards TRIMP (Edwards, 1993) and time spent in the following HR zones: 50–60%, 60–70%, 70–80%, 80–90% and 90–100%/HR_max_. HR_max_ was estimated by the teams’ coach staff, based on the teams’ own procedures. All variables are referred to as the load (i.e., absolute value e.g., meters) and intensity (i.e., value relative to duration e.g., meters per minute) (Gaudino et al., 2015). In further analyses, also the training sessions’ external load and intensity relative to the match load were used (percentage of the match load, where 100% represents the match value).

### Statistical Analyses

Statistical analyses were conducted using IBM SPSS Statistics® software (v28.0, IBM Corporation, Armonk, New York, USA). A linear (LMM) or generalized linear mixed model (GLMM) with repeated measures (microcycles) was used to investigate potential differences between top- and bottom-half teams’ training load and intensity. A group (TH or BH) was included as a fixed effect and a player’s ID as a random effect. In the present study, the Kolmogorov-Smirnov test was used to examine whether the residuals demonstrated normal distribution. The LMM was used for (44/60) variables where their residuals demonstrated normal distribution, as described by West et al. (2022). The GLMM with gamma distribution and log link function was found suitable for the remaining fourteen variables that did not fulfill the assumptions of the LMM (load variables: high-intensity accelerations and decelerations and low-intensity decelerations; intensity variables: distance in speed zone 5 and moderate-intensity accelerations; load relative to the match (percentage of the match load): distance in speed zones 4 and 5, high-intensity accelerations and decelerations and moderate-intensity decelerations; intensity relative to the match (percentage of the match intensity): distance in speed zones 4 and 5 and high-intensity accelerations and decelerations). Results are reported as mean ± standard error and 95% confidence intervals. The significance level was set at *p* < 0.05.

## Results

There were no significant differences in the number of weekly sessions (TH group: 5.8 ± 0.9 [4.1–7.5] and BH group: 5.5 ± 0.9 [3.9–7.2], *p* = 0.176) or total duration (TH group: 486 ± 62 [365–610] min and BH group 514 ± 62 [391–637] min, *p* = 0.087) between groups. However, the average session duration was significantly higher for the BH compared to the TH group (92 ± 7 [78–106] min vs. 85 ± 7 [71–99] min, *p* < 0.001).

Table 1 shows accumulated external and internal load of the groups’ microcycles (including training sessions and the weekly match). There were no significant differences between groups in any variables.

**Table 1:** Top- and bottom-half ranked team’s microcycle training load (mean ± standard error [95% CI]).

External load	Total distance [m]	Speed zone 1 distance [m]	Speed zone 2 distance [m]	Speed zone 3 distance [m]	Speed zone 4 distance [m]	Speed zone 5 distance [m]
Top-half	29057 ± 3290 [22546–35569]	14150 ± 1570 [11044–17256]	9877 ± 1609 [6693–13062]	4006 ± 745 [2532–5481]	747 ± 205 [341–1153]	210 ± 106 [20–400]
Bottom-half	28682 ± 3282 [22188–35177]	13369 ± 1566 [10269–16468]	10412 ± 1602 [7241–13583]	3615 ± 741 [2149–5082]	685 ± 204 [281–1089]	239 ± 105 [31–448]
*p*-value	0.641	0.054	0.220	0.092	0.331	0.209
	Low intensity accelerations [n]	Moderate intensity accelerations [n]	High intensity accelerations [n]	Low intensity decelerations [n]	Moderate intensity decelerations [n]	High intensity decelerations [n]
Top-half	1035 ± 131 [776–1294]	239 ± 41 [157–321]	39 ± 14 [19–72]	1087 ± 134 [850–1389]	238 ± 46 [146–330]	49 ± 18 [24–101]
Bottom-half	1031 ± 131 [768–1285]	216 ± 41 [134–298]	31 ± 11 [15–64]	1035 ± 128 [810–1321]	238 ± 46 [146–329]	46 ± 17 [23–95]
*p*-value	0.794	0.063	0.060	0.105	0.998	0.610
Internal load	Edwards TRIMP [AU]	Time in HR zone 1 [min]	Time in HR zone 2 [min]	Time in HR zone 3 [min]	Time in HR zone 4 [min]	Time in HR zone 5 [min]
Top-half	1247 ± 228 [798–1699]	76 ± 23 [30–121]	96 ± 22 [53–139]	89 ± 20 [49–129]	117 ± 25 [68–166]	46 ± 24 [10–83]
Bottom-half	1279 ± 227 [830–1728]	86 ± 23 [841–131]	100 ± 22 [58–143]	97 ± 20 [57–136]	102 ±25 [54–151]	52 ± 24 [15–88]
*p*-value	0.647	0.176	0.487	0.169	0.064	0.427

Speed zones: 1 (<7 km/h), 2 (7–13 km/h), 3 (13–19 km/h), 4 (19–23 km/h) and 5 (> 23 km/h). Low- (1–2 m/s^2^), moderate- (2–3 m/s^2^) and high-intensity (> 3 m/s^2^) accelerations and low- (−1 to −2) m/s^2^), moderate- (−2 to −3) m/s^2^) and high-intensity (<−3 m/s^2^) decelerations. Heart rate zones (HR_zones_) 1 = 50–60%, 2 = 60–70%, 3 = 70–80%, 4 = 80–90% and 5 = 90–100%/ HR_max_

Table 2 shows the microcycle training intensity of TH and BH groups (including training sessions and the weekly match). The TH group reached higher intensity in total distance per minute, distance in speed zones 1, 3 and 4 per minute, the number of moderate- and high-intensity accelerations and the number of low-intensity accelerations and decelerations per minute compared to the BH group. The only difference between the groups in the internal load was a higher percentage of the time that the TH group spent in HR zone 4.

**Table 2:** Top- and bottom-half ranked team’s microcycle training intensity mean ± standard error and [95% CI].

External load	Total distance (m/min)	Speed zone 1 distance (m/min)	Speed zone 2 distance (m/min)	Speed zone 3 distance (m/min)	Speed zone 4 distance (m/min)	Speed zone 5 distance (m/min)
Top-half	60.3 ± 4.2 [52.0–68.5]	29.3 ± 1.9 [25.6–33.0]	20.6 ± 2.7 [15.4–25.9]	8.3 ± 1.4 [5.6–11.0]	1.5 ± 0.4 [0.7–2.3]	0.4 ± 0.2 [0.1–1.4]
Bottom-half	55.9 ± 4.2 [47.7–64.1]	26.2 ± 1.9 [22.5–29.9]	20.6 ± 2.7 [15.4–25.8]	7.2 ± 1.4 [4.5–9.9]	1.3 ± 0.4 [0.6–2.1]	0.4 ± 0.3 [0.1–1.5]
*p*-value	< 0.001	< 0.001	0.957	0.001	0.024	0.747
	Low intensity accelerations (n/min)	Moderate intensity accelerations (n/min)	High intensity accelerations (n/min)	Low intensity decelerations (n/min)	Moderate intensity decelerations (n/min)	High intensity decelerations (n/min)
Top-half	2.16 ± 0.20 [1.76–2.56]	0.49 ± 0.07 [0.37–0.65]	0.08 ± 0.02 [0.04–0.12]	2.30 ± 0.23 [1.85–2.75]	0.49 ± 0.07 [0.34–0.63]	0.11 ± 0.03 [0.04–0.17]
Bottom-half	2.02 ± 0.20 [1.62–2.41]	0.42 ± 0.06 [0.32–0.56]	0.07 ± 0.02 [0.02–0.11]	2.04 ± 0.23 [1.59–2.49]	0.47 ± 0.07 [0.32–0.61]	0.10 ± 0.03 [0.04–0.16]
*p*-value	0.004	< 0.001	0.003	< 0.001	0.235	0.251
Internal load	Mean heart rate (%/ HRmax)	Time in HRzone 1 (%/ total duration)	Time in HRzone 2 (%/ total duration)	Time in HRzone 3 (%/ total duration)	Time in HRzone 4 (%/ total duration)	Time in HRzone 5 (%/ total duration)
Top-half	70.7 ± 3.3 [64.1–77.2]	16.3 ± 3.8 [8.8–23.8]	19.4 ± 2.8 [13.9–24.9]	18.5 ± 2.9 [12.7–24.3]	22.9 ± 4.3 [14.3–31.5]	9.0 ± 4.4 [1.3–16.6]
Bottom-half	70.0 ± 3.3 [63.4–76.5]	16.8 ± 3.8 [9.4–24.3]	19.7 ± 2.8 [14.2–25.1]	19.2 ± 42.9 [13.4–25.1]	20.0 ± 4.3 [11.5–28.6]	10.4 ± 4.3 [1.9–19.0]
*p*-value	0.368	0.567	0.710	0.278	0.008	0.171

Speed zones: 1 (<7 km/h), 2 (7–13 km/h), 3 (13–19 km/h), 4 (19–23 km/h) and 5 (> 23 km/h). Low- (1–2 m/s^2^), moderate- (2–3 m/s^2^) and high-intensity (> 3 m/s^2^) accelerations and low- (−1 to −2) m/s^2^), moderate- (−2 to −3) m/s^2^) and high-intensity (<−3 m/s^2^) decelerations. Heart rate zones (HR_zones_) 1 = 50–60%, 2 = 60–70%, 3 = 70–80%, 4 = 80–90% and 5 = 90–100%/ HR_max_

**Figure 2:**
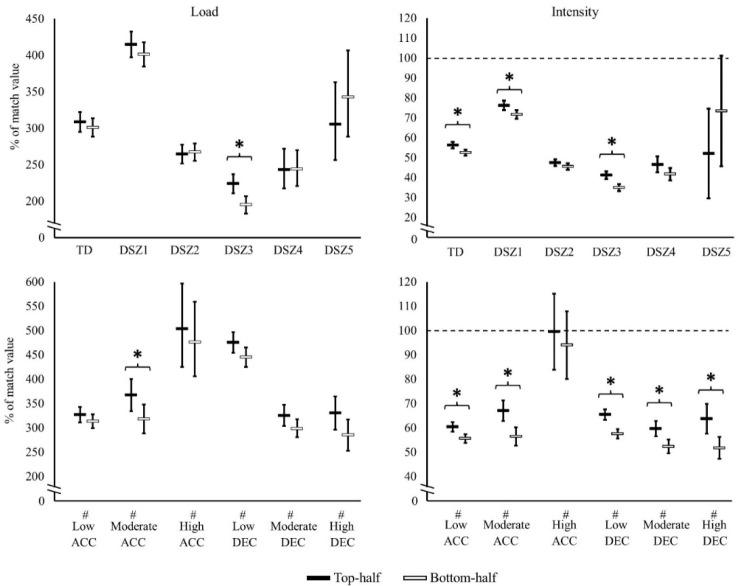
Top- and bottom-half ranked team’s microcycle training load and intensity (mean ± standard error relative to match values (percentages)). TD = total distance, DSZ1–5 = distance on speed zone 1 (<7 km/h), 2 (7–13 km/h), 3 (13–19 km/h), 4 (19–23 km/h) and 5 (> 23 km/h). # low, moderate or high ACC or DEC = number of low- (1–2 m/s^2^), moderate- (2–3 m/s^2^) or high-intensity (> 3 m/s^2^) accelerations or low- (−1 to −2) m/s^2^), moderate- (−2 to −3) m/s^2^) or high-intensity (<−3 m/s^2^) decelerations. The dashed line represents match value (100%). * statistically significant (p < 0.05) difference between top- and bottom-half ranked teams

There were no significant differences in match load variables between TH and BH groups. When players’ training load and intensity were expressed relative to match values, between-group differences were observed as shown in Figure 2. Relative to the match load, TH players achieved higher load in moderate intensity accelerations and in distance covered in speed zone 3 compared to BH players. TH players also achieved higher intensity in total distance, distance in speed zones 1 and 3 and in low- and moderate-intensity accelerations and decelerations, as well as in high-intensity decelerations compared to BH players.

## Discussion

The aim of this study was to quantify the training load of national-level female soccer players during the in-season and to compare the training load and intensity between higher and lower ranked teams in the league. The analyses revealed that the training load of national-level players was quite similar to that reported in previous studies on professional female players, but those professional players achieved a higher training intensity compared to the results presented here (Diaz-Seradilla et al., 2022). There were no significant differences in the weekly external load between this study’s groups. However, TH players consistently achieved a higher intensity, as shown in the following variables: total distance per minute, distance in speed zones 1, 3 and 4 per minute, the number of low-, moderate and high-intensity accelerations, and low-intensity decelerations per minute. The only significant difference in the internal load between the groups was that TH players spent a higher percentage of total time in HR zone 4 compared to BH players. These results show that players from higher ranked teams can generate higher external intensity with a similar internal response (i.e., mean HR) compared to players from lower ranked teams. This suggests that there are differences in training between TH and BH players or that TH players may have better physical qualities inherently.

This study’s Finnish national league players’ microcycle TD load was on average 4–7 km higher and maximal velocity 3–4 km/h higher compared to the findings from professional female players from Spain (Diaz-Seradilla et al., 2022). However, when the average TD intensity is compared between these studies the results reverse. Diaz-Seradilla et al. (2022) reported that the average TD intensity in professional players was between 65 and 72 m/min, while the values in the present study were between 55 and 60 m/min, depending on the group. Vescovi et al. (2021) showed that match TD and distance covered at speed > 16 km/h increased in-line with the competition level and, because match duration is always the same, i.e., 90 minutes, higher intensity values were also obtained from matches in better performing teams/players. Thus, it is logical that higher TD intensity is observed with professional players compared to the present study’s national league players, but because training duration is not constant, the present study’s national players could accumulate a higher TD load. Additionally, direct comparisons with previous studies should be made with caution because of the different speed thresholds delineating high-speed and sprint running.

To the authors’ knowledge, there are no previous studies that have investigated the effect of the league ranking on the training load or intensity. Instead, Houtmeyers et al. (2020) compared microcycle’s accumulated external training load and intensity between the same club’s first-team and U19-team male players and found that there were no major differences in the weekly accumulated training load. Nevertheless, first-team players reached higher external intensity in several variables compared to U19 players. They suggested that U19 players needed longer session duration for development and thus, first-team players were accustomed to shorter training sessions at higher external intensity (Houtmeyers et al., 2020). In the present study, the findings were similar. No differences in accumulated training external or internal load were observed between groups, but TH players reached higher external intensity in several variables. BH players’ individual training sessions were significantly longer (by ~7 min), but there were no differences in the number of weekly sessions or total duration. This seven-minute difference is likely not meaningful physiologically, but it does impact the calculation of intensity and, thus, it may explain some part of the difference in the external intensity variables between groups, as reported in Houtmeyers et al. (2020).

A more meaningful factor to explain the difference between playing levels could be TH players’ better physical qualities. In the present study, TH players’ external intensity was significantly higher in several variables compared to BH players. Simultaneously, the only difference in internal intensity was that TH players spent a higher percentage of total time in HR zone 4. Thus, TH players could generate higher external intensity in multiple variables while maintaining similar internal intensity (e.g., HR_mean_) compared to BH players. A logical explanation for this finding could be that players of higher ranked teams have better physical qualities and, thus, they are able to work at higher external intensity with similar internal response compared to players from lower ranked teams. This proposal remains a speculation because player’s physical qualities were not evaluated in this study, although previous studies have shown that levels of physical qualities in female soccer players increase along with the competition level (Haugen et al., 2012, 2014). Players’ physical qualities can be improved either soccer-specifically, e.g., small-sided games, or by isolated fitness training, such as high-intensity interval training (Nayıroğlu et al., 2022).

Previous studies have shown that the external load and intensity in match-play also increase in-line with the competition level (Vescovi et al., 2021), but in the present study there were no differences in the match external load nor intensity between groups. The match is the microcycle’s dominant performance during the in-season and, thus, it is reasonable to compare the microcycle load and intensity to match values, as previously reported in male players (Stevens et al., 2017). TH players’ microcycle external training intensity relative to match intensity was higher than in BH players in the following variables: TD, distance covered in speed zones 1 and 3, the number of high-intensity decelerations, and low- and moderate-intensity accelerations and decelerations. This indicates that TH players’ external training intensity is closer to match demands in these variables than for BH players, which could contribute to better preparation of TH players for matches. In the accumulated microcyle external load relative to match, the only significant differences between groups were TH players’ greater distance covered in speed zone 3 and a higher number of moderate intensity accelerations. This confirms that, regardless of whether the training load is reported as an absolute value or relative to the match load, the team’s level is not greatly influential, but external intensity variables differ between top- and bottom-half ranked teams.

In the present study, microcycle absolute total distance and distance covered in speed zones 1–5 was ~200–400% of the match load and the number of different intensity accelerations and decelerations was ~300–500% of the match load. Compared to findings from elite-level male players, the microcycle load relative to match results is noticeably similar: approximately threefold total distance and the number of high-intensity decelerations (Stevens et al., 2017). In the present study, a greater number of high-intensity accelerations relative to the match load was reported compared to elite-male players and there was also a tendency for greater high-intensity running compared to elite male players (Stevens et al., 2017). These findings indicate that training load relative to national-level soccer players’ own capacity (match performance) are similar or even slightly higher compared to findings from elite male players during the in-season. Female players’ higher training load relative to the match could be because females exhibit less muscle fatigability and faster recovery during endurance exercise compared to males (Bassett et al., 2020). However, direct comparisons should not be made, or at least made with caution, because of different speed thresholds between sexes (Stevens et al., 2017).

The biggest strength of this study was that it combined data from six different teams from the same league, while previous studies had typically observed only one single team (Costa et al., 2022). This method may help better generalize results for national-level female soccer players and provide normative data, compared to relying on data from a single team. The involvement of six teams also made it possible to compare training load between top-half and bottom-half ranked teams, which is a novel approach in training load research.

Even though there have been several training load studies conducted over similar or even shorter duration (Costa et al., 2022), the most important limitation of the present study was the short observation period. First, there was a relatively high number of data exclusion since only a limited number of players per team were selected to play in the weekly match. This led to exclusion of all players who did not play at least 60 min in the match of that week. Nevertheless, this criterion was needed to calculate training load and intensity values relative to the match and, thus, such a methodological decision was made. Secondly, the observation period was only three weeks at the beginning of the in-season, which might not have accurately reflected the playing standard that led to the league ranking at the end of the season. However, the difference in average points per match between the groups was clear and consistent throughout the entire season and, thus, the observation period was reflective of the end of the season ranking. Also, in the present study, different playing positions were not separated because of the limited sample size. Previous studies have shown that the playing position affects the match load (Mäkiniemi et al., 2023; Romero-Moraleda et al., 2021), while there are mixed findings in training load (Doyle et al., 2022; Romero-Moraleda et al., 2021). In the present study, the proportion of different playing positions was similar between groups, suggesting that comparisons made at a whole-team level were justifiable. The final limitation was HR_max_ assessment, which was not standardized in this study. Some teams used the maximal HR value measured from a maximal test, while other teams used the highest HR value measured during training sessions or matches.

The present study showed that higher ranked teams reached higher training external intensity than lower ranked teams. As there are no similar studies performed with female players previously, the present study’s results cannot be directly compared to previous findings. Thus, in future, studies should use several teams and collect data across a full season to investigate differences in training load and intensity between teams of different competitive levels, playing positions and season phases.

## Conclusions

The main finding of this study is that players from top-half ranked teams reached higher microcycle (weekly) external intensity, both average and relative to the match, compared to players from bottom-half ranked teams. Thus, it is possible that training prepares players from higher-ranked teams better to match demands compared to players from lower-ranked teams. However, there were no major differences in the microcycle’s internal load or intensity between groups. These results show that players from higher ranked teams can generate higher external intensity from a similar internal response (i.e., mean HR) compared to players from lower ranked teams. This suggests that there are differences in training formats between top- and bottom-half ranked teams or players from top-half teams may have better physical qualities.

## Practical Implications

Coaches of less successful teams should aim to increase training external intensity progressively to possibly prepare players better to match demands. From a coaching perspective, one solution could be to vary training formats (small-, medium- and large-sided-games) systematically to generate higher external intensity for selected variables depending on the format. However, increasing in-season training external intensity would also increase internal load/intensity, which could lead to suboptimal preparation for the match in the short-term. Thus, coaches, especially from less successful teams, should also focus on improving players’ physical qualities in the long-term, which would enable players to generate higher external load/intensity with similar internal response to those of higher level players.
